# Inflammatory response triggered by avian hepatitis E virus *in vivo* and *in vitro*

**DOI:** 10.3389/fimmu.2023.1161665

**Published:** 2023-03-30

**Authors:** Yawen Zhang, Zengna Chi, Zhizhong Cui, Shuang Chang, Yixin Wang, Peng Zhao

**Affiliations:** ^1^ College of Veterinary Medicine, Shandong Agricultural University, Tai’an, Shandong, China; ^2^ Shandong Provincial Key Laboratory of Animal Biotechnology and Disease Control and Prevention, Shandong Agricultural University, Tai’an, Shandong, China; ^3^ Shandong Provincial Engineering Technology Research Center of Animal Disease Control and Prevention, Shandong Agricultural University, Tai’an, Shandong, China

**Keywords:** Avian hepatitis E virus, liver injury, immune response, inflammation, cell culture

## Abstract

Hepatitis E virus (HEV) is relevant to public health worldwide, and it affects a variety of animals. Big liver and spleen disease (BLS) and hepatitis-splenomegaly syndrome (HSS) associated with avian HEV (aHEV) were first reported in 1988 and in 1991, respectively. Here, cell culture–adapted aHEV genotype 3 strain, YT-aHEV (YT strain), a typical genotype isolated in China, was used for basic and applied research. We evaluated liver injury during the early stages of infection caused by the YT strain *in vivo*. Both *in vivo and in vitro* experimental data demonstrated that viral infection induces innate immunity, with mRNA expression levels of two key inflammatory factors, interleukin-1β (IL-1β) and IL-18, significantly upregulated. The YT strain infection was associated with the activation of Toll-like receptors (TLRs), nuclear factor kappa B (NF-κB), caspase-1, and NOD-like receptors (NLRs) in the liver and primary hepatocellular carcinoma epithelial cells (LMH). Moreover, inhibiting c-Jun N-terminal kinase, extracellular signal–regulated kinase (ERK1 or 2), P38, NF-κB, or caspase-1 activity has different effects on NLRs, and there is a mutual regulatory relationship between these signaling pathways. The results show that SB 203580, U0126, and VX-765 inhibited IL-1β and IL-18 induced by the YT strain, whereas Pyrrolidinedithiocarbamate (PDTC) had no significant effect on the activity of IL-1β and IL-18. Pretreatment of cells with SP600125 had an inhibitory effect on IL-18 but not on IL-1β. The analysis of inhibition results suggests that there is a connection between Mitogen-activated protein kinase (MAPK), NF-κB, and the NLRs signaling pathways. This study explains the relationship between signaling pathway activation (TLRs, NF-κB, MAPK, and NLR–caspase-1) and viral-associated inflammation caused by YT strain infection, which will help to dynamic interaction between aHEV and host innate immunity.

## 1 Introduction

Hepatitis viruses can be categorized into five types: A, B, C, D, and E viruses (HAV, HBV, HCV, HDV, and HEV, respectively). Hepatitis E virus (HEV) belongs to the *Hepeviridae* family, which has two genera: *Orthohepevirus* and *Piscihepevirus*. The *Orthohepevirus* genus has four species (A, B, C, and D) and is known to infect a wide range of mammalian species, including humans, swine, wild boar, chickens, rats, ferrets, rabbits, mongoose, camels, cows, and bats (1). HEV infection–induced liver diseases are intricately related to the dysregulation of immune responses, such as severe liver inflammation. Several researchers have reported significant progress in the molecular biology, diagnosis, epidemiology, and clinical features of HEV infections in various species in recent years.

aHEV is a positive-sense, single-stranded RNA virus that belongs to the *Orthohepevirus B* family. The full-length aHEV genome is approximately 6.6 kb long and 600 bp shorter than mammalian HEV. It begins with a short 5′ non-coding region (NCR), followed by three open reading frames (ORFs), and a short 3′NCR ending with a poly(A) tract (2). Approximately 30%~48% of the aHEV genome is the same as the mammalian HEV genome. aHEV is the main causative agent of big liver and spleen disease (BLS) and hepatitis-splenomegaly syndrome (HSS). To date, aHEV has been detected in chickens in many countries, including the United States, Hungary, Canada, Australia, Spain, China, Korea, Poland, and Israel, and the cross-border regions of Austria and Czechia (3–10). aHEV infection is common in chicken flocks; however, there are no effective measures, commercial vaccines, or drugs available for preventing the disease in chickens. Diagnosis of the clinical manifestation of aHEV is primarily based on the detection of specific viral RNA. Our understanding of its pathogenesis is hampered by the lack of an efficiently propagated cell culture system, as the virus can only proliferate within living organisms or animals (11).

The liver is an important immunological organ and is enriched with innate immune cells [macrophages, innate lymphoid cells, mucosal-associated invariant T (MAIT) cells] that can rapidly activate the immune response to liver diseases, infections, or tissue damage (12). Inflammatory responses are important components of the innate immune response during viral infections. The liver is also the primary target organ following aHEV infection (13). Since 2016, hepatic rupture hemorrhage syndrome (HRHS) has emerged in layer and broiler breeder hens in several provinces of China. The liver exhibits hemorrhage and swelling (14). In 2020, diseases similar to HRHS caused by the aHEV occurred in a Roman Brown layer farm in Jiangxi province (15). Moreover, a genetically novel aHEV was reported in Jiangxi province, China, in 2020. The chickens had severe hepatosplenomegaly syndrome, many bleeding spots, and apparent bleeding clots in the livers (16). In 2022, a study described cases of aHEV associated with hepatosplenomegaly, rupture, and hemorrhage from several cities in Shandong (17).

Underlying liver disease, Toll-like receptor (TLR) signaling, or inflammasome activation initiates inflammatory response in the liver (13). Pathogen-associated molecular patterns (PAMPs) activate TLRs, as pattern recognition receptors (PRRs), which function as the first barrier of natural immunity *via* downstream related molecules. Recent studies have indicated that TLR signaling plays an important role in triggering antiviral and inflammatory responses (18). TLR ligands activate various downstream intracellular signaling cascades, including NF-κB and MAPK (19), two well-known signaling pathways controlling in inflammation and apoptosis. The MAPK cascades has four prototype members: extracellular signal–regulated kinase (ERK) 1/2, p38, the c-Jun N-terminal kinase (JNK/SAPK), and ERK5 (20, 21). In addition to these pathways, inflammasome activation is a hallmark for the pathogenesis and progression of many inflammatory diseases (22). Inflammasomes are intracellular multiprotein complexes found in both parenchymal and non-parenchymal liver cells that activate caspase-1 and release IL-1β and IL-18 in response to cellular danger signals (22).

In this study, specific pathogen–free (SPF) chick embryos were infected with the aHEV *via* intravenous inoculation to determine the specificity of the liver damage caused by aHEV infection. We also conducted an *in vitro* viral infection experiment with LMH cells and confirmed that aHEV infection induces an immune response. The roles of relevant signaling pathways and important genes during the aHEV (YT strain) infection were investigated both *in vivo* and *in vitro*. Interactions between different pathways were subsequently explored using inhibitors of signaling pathways.

## 2 Materials and methods

### 2.1 Cell line and virus

The chicken liver cell line (LMH; ATCC^®^ CRL­2117™; organism: *Gallus gallus*, chicken; strain: leghorn; tissue: liver; disease: hepatocellular carcinoma; cell type: chemically induced; morphology: epithelial; growth properties: adherent). The LMH cell was cultured in Dulbecco’s modified Eagle medium/F-12 with 10% fetal bovine serum (FBS) and 1% penicillin (100 U/ml)–streptomycin (10 mg/ml) in a humidified incubator at 37°C with 5% CO_2._

YT-aHEV (YT strain; GenBank No. MZ736614), a strain of aHEV, was originally isolated from a liver sample from white broiler breeders with liver hemorrhage and swelling and was propagated in LMH cells following stable replication in LMH cells (23).

### 2.2 Viral infection *in vivo*

Twenty SPF chicken embryos (SPAFAS poultry company, China) were intravenously inoculated with 100 Tissue culture infective dose (TCID_50_) of the aHEV infectious stock. Ten SPF chicken embryos inoculated with phosphate-buffered saline (PBS; 0.01 M, pH 7.2) served as the control group. Serum samples were tested for anti-aHEV antibodies using specialized commercial aHEV antibody kits (IDvet, France). Lymphocytes, liver tissue, and fecal samples from the same positive individuals were collected immediately and monitored for aHEV RNA (**Table 1**). Liver tissue samples were collected at each necropsy, homogenized in 10% (w/v) sterile PBS (0.01 M, pH 7.2), and centrifuged at 13,000*g* for 3 min at 4°C to extraction RNA.

**Table 1:** Detection of HEV RNA and antibody.

Group	Sample			Antibody Serum
	Liver	Feces	Lymphocytes	
Infection	13/14	13/14	12/14	0/14
Control	0/7	0/7	0/7	0/7

### 2.3 Pathology and histopathology evaluations

Gross pathological lesions in the liver were evaluated and photographed. All chickens were dissected, and the liver index was calculated according to the formula: (liver weight/body weight) × 100%. Liver samples were fixed in 10% neutral-buffered formalin and processed for routine histological examinations.

### 2.4 Determination of ALT and AST concentrations and anti-HEV antibodies

The plasma concentrations of alanine aminotransferase (ALT) and aspartate aminotransferase (AST) were measured on the day of collection using a standard procedure following the manufacturer’s instruction. ALT and AST commercial reagent kits were purchased from Nanjing Jiancheng Bioengineering Institute (Nanjing, Jiangsu, China).

Anti-aHEV IgG antibodies were detected in the serum samples using Avian Hepatitis E Indirect ELISA (IDvet, France). This indirect ELISA diagnostic kit is designed to detect antibodies directed against aHEV (aHEV) in chicken that was developed for the chicken serum or plasma. Serum samples were diluted 500 times with the kit diluent and used in the assay. The entire reaction was carried out at 37°C, and the final absorbance values were read and recorded at 450 nm.

### 2.5 ELISA for evaluating IL-18 and IL-1β levels

The expression levels of IL-18 and IL-1β in plasma and cell cultures were determined using Enzyme linked immunosorbent assay (ELISA). IL-18 and IL-1β ELISA kits were purchased from Senbeijia Biological Technology (Nanjing, Jiangsu, China).

### 2.6 Viral infection and measurement of innate immunity in LMH cells

LMH cells were seeded in 24-well cell culture plates and infected with a dose (300 TCID_50_) of YT strain. The supernatant above the infected cells was discarded at 4 h post-infection (hpi), and medium without FBS was added to the cells. Supernatants from the infected cells were collected separately at 1, 2, 3, 4, 5, and 6 days post-infection (dpi). Genes associated with innate immunity in LMH cells were detected and analyzed. Primers used for real-time polymerase chain reaction (PCR) shown in **Table 2**. Some primer pair sequences were obtained from the literature (24).

**Table 2:** Primers used for real-time PCR.

Target gene	Primer sequence	GenBank accession no.	Product size (bp)
MDA5	F: 5′-TGAAAGCCTTGCAGATGACTTA-3′	NM_001193638.2	134
	R: 5′-GCTGTTTAAATCCTCCGTTAC-3′		
MAVs	F: 5′-CACCCACGAGGTCCATGTG-3′	NM_001012893.2	88
	R: 5′-TGCTTCATCTGGGACATCATTG-3′		
IFN-α	F: 5′-CTGCTGCTCACGCTCCTTCTG-3′	NM_205427.1	185
	R: 5′-GTGTCCAGGATGGTGTCGTTGAAG-3′		
IL-1β	F: 5′-TGCCTGCAGAAGAAGCCTCG-3′	NM_204524.2	138
	R: 5′-CTCCGCAGCAGTTTGGTCAT-3′		
TLR4	F: 5′- CATCCCAACCCAACCACAGTAGC-3′	NM_001030693.2	119
	R: 5′-CCACTGAGCAGCACCAATGAGTAG-3′		
TLR5	F: 5′- ACTCCCTTCCTTCCCACATCTGAC-3′	NM_001398059.1	87
	R: 5′-TGTGTTGCTACTATTGCCGTGTGAG-3′		
TLR15	F: 5′-GATGGGCTGTGGTATGTGAGAATGG-3′	NM_001398238.1	145
	R: 5’-TCAGTAGATGCTCCTTCGTCCAGTC-3′		
TLR21	F: 5′-TCTCACAGGCGGAGGTCTTCAC-3′	NM_001030558.3	116
	R: 5′-GCGAGGTTGGATGTCAGAGATGTC-3′		
NLRP3	F: 5′-GCTCCTTGCGTGCTCTAAGACC-3′	NM_001348947.2	150
	R: 5′-TTGTGCTTCCAGATGCCGTCAG-3′		
NLRC3	F: 5′-GAGGAAGCGATGAAGAACGAGAGC-3′	XM_015294675.4	132
	R: 5′-GTTGTAAGTGAGGCAGTTGGAGAGG-3′		
NLRC5	F: 5′-CAGGTTGGCAGAGGAACTTGTCAG-3′	NM_001318435.2	128
	R: 5′-AACAGAAGGAGAAGCACAGTCTTGG-3′		
NLRX1	F: 5′-CTCAGGCGGTGTCACATTCAGTC-3′	XM_003642592.6	88
	R: 5′-GGTTTGATGGGACGGTCACTTCTC-3′		
NLRP1L	F: 5′- GGCTGAGTGATGAGTTGGAGAAGTG-3′	XM_040705907.1	141
	R: 5′-ACGCTCTAAACTTTCCGCTGACTG-3′		
Caspase-1	F: 5′-CAAGAGTAATGGGACCACGGACATC-3′	XM_015295935.4	119
	R: 5′-CACGGCAGCACTGGATAATGACC-3′		
MYD88	F: 5′-CTGGTGACTGTGGAGCAAGGAAAG-3′	NM_001030962.5	124
	R: 5′-ACCCAACCTAAAGCACTGACATCTG-3′		
IL-10	F: 5′-GCTGTCACCGCTTCTTCACCTG-3′	NM_001004414.4	98
	R: 5′-GGCTTTGTAGATCCCGTTCTCATCC-3′		
IL-4	F: 5′-GAAAGTCCTGGGATACGGAGAAACG-3′	NM_001007079.2	82
	R: 5′-ACAGTGGTAGGAGGCAGATGGTG-3′		
IL-6	F: 5′-TCAGAGGCGAATGTTGGTGGAATG-3′	NM_001007079.2	90
	R: 5′-GCTGCCATCTGTCACACGGTAAC-3′		
TGF-β	F: 5′-CCGACACGCAGTACACCAAGG-3′	NM_001318456.1	125
	R: 5′-ATTCCGGCCCACGTAGTAAATGATG-3′		

F, forward; R, reverse.

### 2.7 Measurement of aHEV growth in LMH cells

LMH cells were seeded in 24-well cell culture plates. These cells were infected with aHEV, and the cells and cell culture supernatants were collected at 1, 2, 3, 4, 5, and 6 dpi. A SYBR Green (TaKaRa) real-time reverse transcription PCR (RT-PCR) assay was developed to detect aHEV-RNA. Primer sets targeting a 113-bp region of the known ORF2 gene of the YT strain (genotype 3). Primer sets: qH-F1 (5′-AGATATGAATTCTATTACAT-3′) and qH-R1 (5′-GCCAGCCGTTATTCTTGTAT-3′). The copy number was calculated using the following formula: Ct (Cycle threshold) = −3.112 4 lg (number of copies) + 37.13.

### 2.8 Viral infection–induced inflammation response in LMH cells

LMH cells were infected with YT strain at doses (TCID_50_) of 150 and 300. The supernatant of the infected cells was collected, and IL-1β and IL-18 were detected at 1, 2, 4, and 6 hpi using ELISA. Infected LMH cells were also collected at 1, 2, 3, 4, 5, and 6 dpi and analyzed using qRT-PCR to detect genes related to inflammation-associated signaling pathways in LMH cells. Gene sequences were obtained from the NCBI database, and primers were designed using the Primer-BLAST tool and specificity by Shanghai Sangon Biological Engineering Technology.

### 2.9 Preliminary exploration of the optimal concentration of inhibitor

LMH cells were inoculated to 96-well plates, and cell fusion reached 80% before incubation with inhibitors. Adezmapimod (SB 203580) is a selective and ATP-competitive p38 MAPK inhibitor (25). SP600125 is an ATP-competitive JNK inhibitor (26). U0126 is a potent, non-ATP competitive and selective MEK1 and MEK2 inhibitor (27). PDTC is a selective and blood–brain barrier permeable NF-κB inhibitor (28). VX-765 is a potent and selective inhibitor of IL-converting enzyme/caspase-1 (29). SB 203580, SP600125, U0126, and PDTC were purchased from Beyotime Biotechnology. The VX-765 was purchased from ABclonal Technology Co., Ltd. The inhibitors were diluted with Dimethyl sulfoxide (DMSO) according to the manufacturers’ instructions and previous studies (30).

CCK-8 Cell Counting Kit (Vazyme, China) was used to assess the toxic effects of different concentrations of inhibitors on cells. Wells without the inhibitors were used as controls. The inhibitors were incubated with LMH cells for 2 h and then removed. The cells were then washed with PBS (0.01 M, PH 7.2), and 100 μl of culture medium was added together with 10 μl of CCK-8 solution into each well of cell culture plate for 1 h under 37 °C. During this addition, air bubbles should be avoided. The absorbance value was measured at 450 nm using an enzyme marker (Molecular Devices, SpectraMax i3x). A higher absorbance value means that more methanogenic dye is generated and more live cells are present. The maximum safe concentration of different inhibitors was determined on the basis of the measured results of the cell viability assay and used as the treatment concentration for subsequent experiments.

### 2.10 Signaling pathway inhibition assay

LMH cells were treated with SB 203580 (30 μmol/L), SP600125 (50 μmol/L), U0126 (10 μmol/L), PDTC (5 μmol/L), and VX-765 (10 μmol/L) for 2 h. The cells were then washed once and infected with aHEV at 300 TCID_50_. After treatment, cells without aHEV infection were used as controls. Cells were collected at 6 hpi and analyzed using qPCR.

### 2.11 RNA extraction and quantitative real-time PCR

Total RNAs was extracted from cells and liver samples using an Omega total RNA kit, and cDNA was synthesized using the RT SuperMix for qPCR (gDNA eraser) kit (Takara Biomedical Technology, DaLian, China) according to the manufacturer’s protocol. Real-time PCR was performed using SYBR qPCR MasterMix (AG, Nanjing, China) in an Q5 Real-Time PCR System (Applied Biosystems, CA, USA) according to the manufacturer’s protocol. Gene expression levels were normalized to that of the housekeeping gene *β-actin*. The changes in gene expression levels were calculated using the threshold cycle (2^−ΔΔCT^) method (31).

### 2.12 Statistical analysis

The data were analyzed using Prism 8.0.2 software (GraphPad Software, La Jolla, CA). Significance levels were set as follow: *P = 0.05; **P = 0.01; ***P = 0.001.

## 3 Results

### 3.1 Detection of aHEV RNA and aHEV IgG from chickens

aHEV RNA was detected in serum, fecal, bile, and liver tissue samples from chickens in both the intravenous inoculation and normal saline groups. All control chickens remained negative throughout the experiment. Fecal viral shedding and virus-positive liver samples were detected in 13 of the 14 inoculated chickens. Plasma samples were positive for aHEV RNA in 12 of the 14 chickens. This indicates that these analyses can be used as an immunological indicators of HEV infection. Anti-aHEV antibodies were detected in serum samples using ELISAs, and the results showed that all samples from both the infected and normal saline control groups were negative for antibodies.

### 3.2 HEV infection leads to liver injury

**Figure 1:**
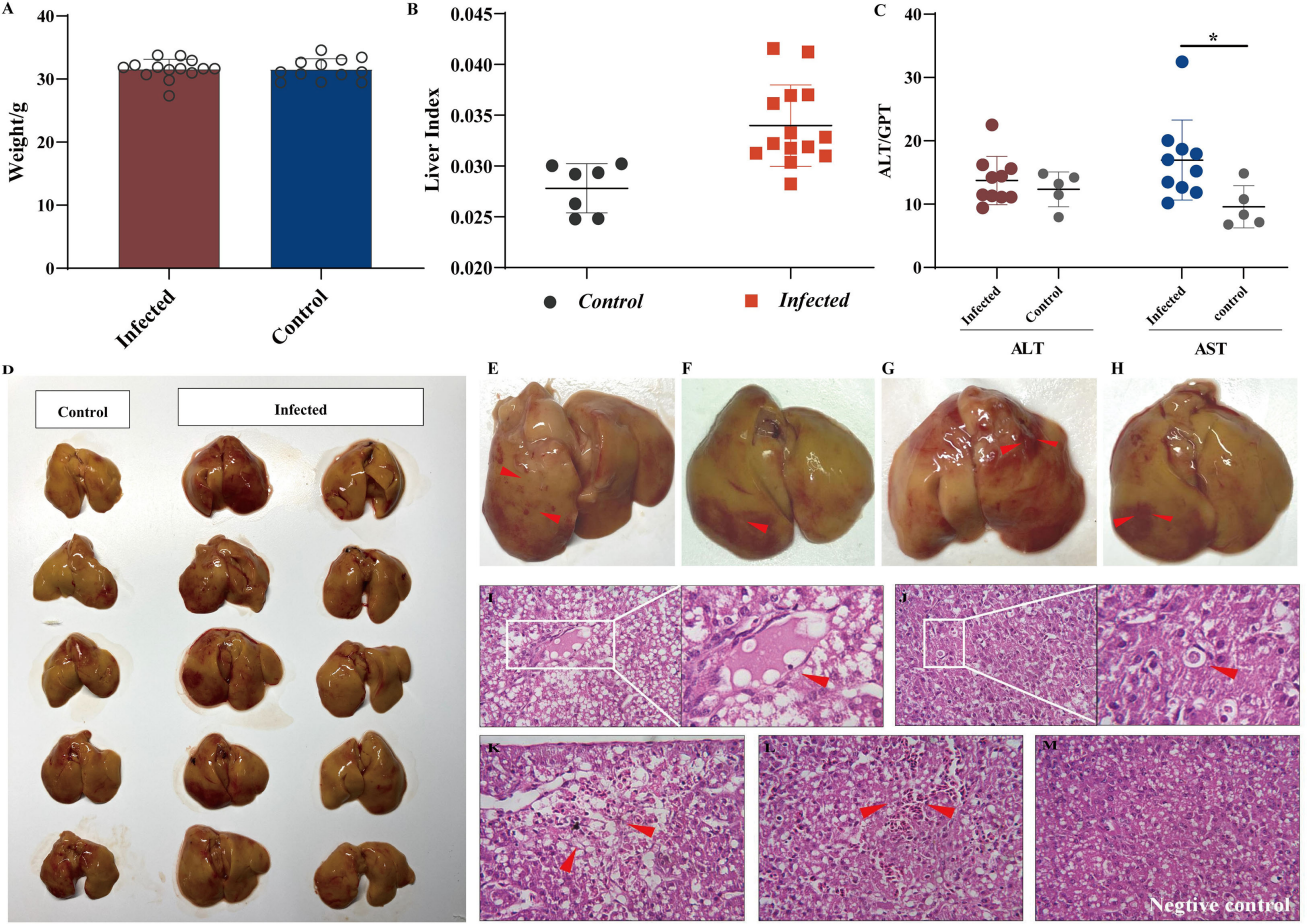
Liver injury analysis in chicken infected with aHEV. **(A)** Analysis of weight data. **(B)** Liver to body weight ratio was calculated by (liver weight)/(body weight) **×** 100%. **(C)** ALT levels and AST levels in SPF chickens experimentally infected with YT strain or saline. **(D)** Gross lesions in the liver in the negative control group: no obvious pathological signs were seen, and the liver was normal in color. **(E–H)** Livers from the inoculated chickens showed enlargement and subperitoneal hemorrhage. Livers from the infected group exhibited severe lesions (red arrow) including thrombosis **(I)**, apoptotic bodies **(J)**, hemorrhage and hepatocellular necrosis **(K)**, and congestion **(L)**. **(M)** Liver from negative control group showing no visible lesions. Tissues were stained with hematoxylin and eosin (stained sections were imaged at ×40). Symbols indicate comparison vs. control group; *P < 0.05.

The body weight data presented in **Figure 1A** indicate that the viral infection had no significant influence on chicken embryonic development. This study aimed to observe early lesions focusing on the liver, and all chicks were necropsied on the first day after hatching. We calculated the liver indices for all individuals to assess whether aHEV-infected livers were enlarged. The liver indices of all chickens (liver weight/body weight × 100%) were calculated and are presented in **Figure 1B**. The liver index of the inoculated chickens was higher than that of the control group, indicating that the livers of SPF chickens were enlarged after viral infection. As a result of the gross pathological lesions and liver histopathology, we evaluated the enzymes ALT and AST, which are indicators of liver damage. ALT activity showed no significant change (**Figure 1B**) when compared to the control, whereas AST levels increased (**Figure 1C**). Liver lesions are shown in **Figure 1D**. The livers of the inoculated chickens were significantly thickened and swollen when compared to the control group. The infected livers became greasy; the edge was obtuse and round, and there were obvious bleeding spots on the liver surface, similar to clinical cases (**Figures 1E–H**). Histopathological observations supported the above phenomenon and showed that the YT strain caused liver damage, such as congestion, hepatic steatosis, and apoptotic bodies (**Figures 1I–M**). No hepatic lesions were observed in the control group.

### 3.3 Host innate immune response induced by YT strain

**Figure 2:**
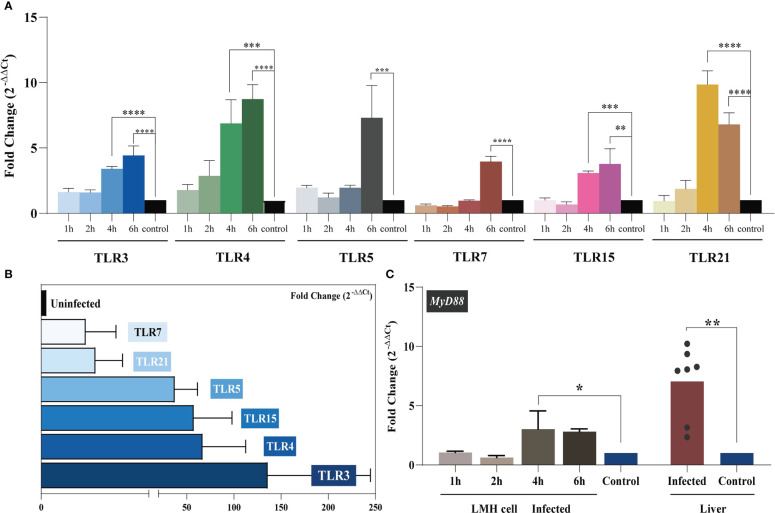
TLR mRNA expression after YT strain infection *in vitro* and *in vivo*. **(A)** The relative mRNA levels of TLRs in LMH cells. The mRNA level of TLRs increased as infection time increased. The infected group and the control group are represented in blue, green, gray, red, pink, yellow, and black, respectively. **(B)** The relative mRNA levels of TLRs in liver tissue. **(C)** MyD88 expression in LMH cells and SPF chickens. Symbols indicate comparison versus control group; *P < 0.05, **P < 0.01, ***P < 0.001 and ****P < 0.0001.

**Figure 3:**
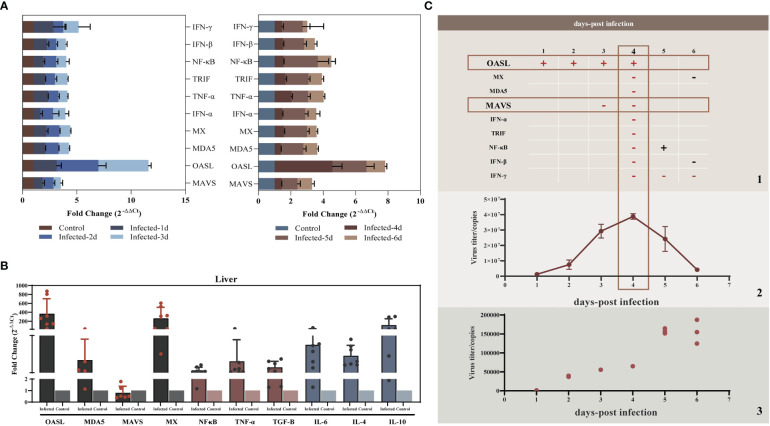
YT strain–triggered innate immune responses *in vitro* and *in vivo*. **(A)** Transcriptional levels of *MDA5*, *OASL*, *MX*, *MAVS*, *IFN-α*, *TRIF*, *NF-κB*, *IFN-β*, and *TNF-α* associated with innate immunity in LMH cell were analyzed at 24, 48, 72, 96, 120, and 144 hpi. **(B)** Transcriptional levels of MDA5, OASL, MX, MAVS, NF-κB, and TNF-α in SPF chicken liver. **(C)** Association between viral replication trends and antiviral proteins. (C.1) The red rectangles mark the two antiviral proteins with significant differences of upregulated (OASL) or downregulated (MAVS) expression levels for the corresponding time points. The “+” represent upregulated, and the “−” represents downregulated. (C.2) The copy number of viral RNA that was present in the LMH cell. (C.3) The copy number of viral RNA that was present in the cell culture supernatant.

Studies have shown that liver injury due to viral hepatitis is caused by the host immune response to viral proteins expressed by infected hepatocytes rather than by the direct cytopathic effects of viruses (32). To elucidate the innate immunity induced by YT strain infection, LMH cells were infected with the YT strain. Real-time PCR was then used to examine the mRNA expression levels of several antiviral proteins or cytokines associated with innate immunity in infected cells. The levels of 2'-5'-oligoadenylate synthase-like protein (OASL) in the YT strain–infected LMH cells increased from 24 to 96 hpi when compared with the uninfected LMH cells (**Figure 2**). It is worth noting that the antiviral signaling protein Mitochondrial Antiviral Signaling Protein (MAVS) decreased mRNA level in cells after infection with YT strain. Other antiviral proteins and cytokines such as Melanoma Differentiation-Associated Protein 5 (MDA5), TIR-domain-containing adaptor-inducing interferon-β (TRIF), antiviral interferon-α (IFN-α), IFN-β, IFN-γ, NF-κB, and tumor necrosis factor–α (TNF-α), were not significantly different in either the infected cells or the uninfected cells (**Figure 3A**). To confirm the conclusions *in vivo*, the expression levels of four kinds of antiviral proteins, NF-κB, and TNF-α in the livers from YT strain–infected chickens were analyzed. As illustrated in **Figure 3B**, infection with the YT strain upregulated the expression level of OASL, recording a 300-fold increase, whereas the level of MAVS was downregulated compared to that in the normal liver. These data demonstrate that the YT strain can induce high levels of OASL and inhibit MAVS expression *in vitro* or *in vivo*. Following infection with the YT strain, MX level was significantly elevated in the *in vivo* study when compared with that in the *in vitro* study. There is always a close relationship between viral infection, replication, and host innate immunity. We therefore depicted the growth curves of the intracellular and extracellular viruses at different post-infection times. Over time, the level of intracellular viruses gradually increased, reaching a peak at 4 dpi, indicating that the antiviral proteins and associated cytokines were all downregulated. On the days 5 and 6 after infection, the intracellular virus content began to decrease, whereas the extracellular virus content increased significantly, indicating that the majority of viruses were released extracellularly (**Figure 3C**).

### 3.4 TLRs mediate inflammatory signaling pathways

During infection, PRRs drive innate immune responses to pathogen-derived mediators (PAMPs). TLRs are PRRs that are expressed by various immune cells. TLR-mediated recognition of pathogens by innate immune cells plays an important role in the induction of pro-inflammatory immune responses required to clear the infection (33). We analyzed the expression level of each avian TLR *in vitro* and *in vivo* with regard to the level of MYD88 signaling. LMH cells were treated with 300 TCID_50_ of the YT strain, and intracellular TLR expression was assessed at 1, 2, 4, and 6 hpi. The results are illustrated in **Figure 2A**. Over time, the levels of TLRs progressively increased. For all six TLRs, the mRNA levels were significantly higher at 6 hpi compared to the control group. The *in vivo* assay results supported these *in vitro* results. Liver TLR 3, 4, 5, 7, 15, and 21 mRNA expression levels were related to YT strain infection. Increased levels of TLR3 in aHEV–positive individuals were the most significant (up to a hundredfold). Collectively, these results suggested that TLRs participate in the initiation of inflammatory processes.

### 3.5 YT strain–induced inflammatory response *in vitro* and *in vivo*

**Figure 4:**
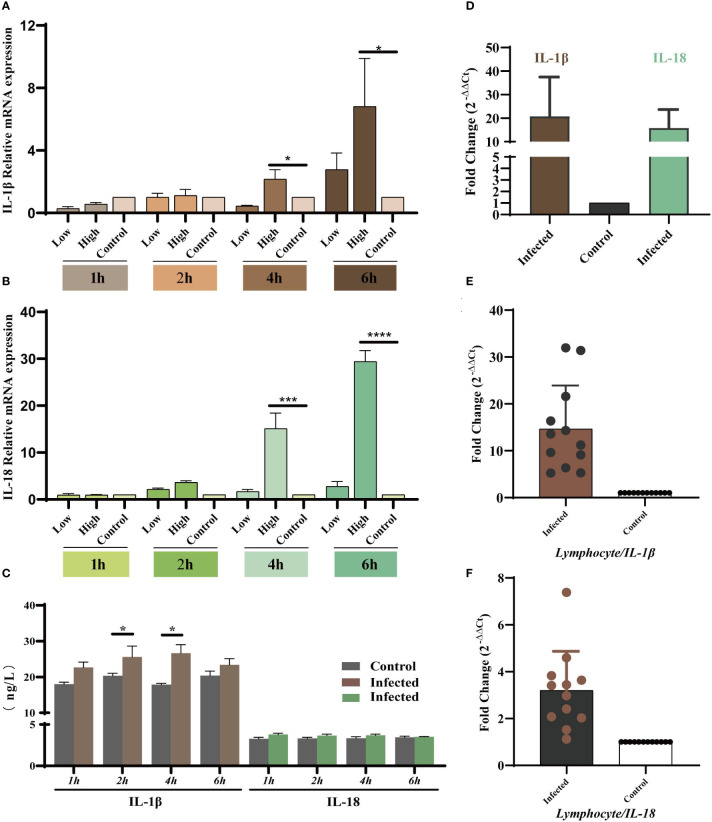
YT strain infection increases levels of IL-1β and IL-18 mRNA expression and secretion *in vitro* and *in vivo*. **(A)** IL-1β gene expression levels at different times post-infection measured using qRT-PCR assays. **(B)** IL-18 gene expression levels at different times post-infection measured using qRT-PCR assays. **(C)** IL-1β and IL-18 levels at different times post-infection detected using ELISA. **(D)** The mRNA expression levels of IL-1β and IL-18 in the liver tissue detected using qPCR. **(E, F)** The mRNA expression levels of IL-1β and IL-18 lymphocytes compared with control group. Symbols indicate comparison vs. control group; *P < 0.05, ***P < 0.001 and ****P < 0.0001.

As the key players in the regulation of inflammatory processes, the expression levels of IL-1β and IL-18 were measured *in vivo* and *in vitro* to explore the relationship between YT strain infection and inflammation. LMH cells were infected with both high and low doses of the virus, and the cells and supernatants were collected at 1, 2, 4, and 6 hpi. The results showed that IL-1β and IL-18 mRNA expression level was upregulated in a dose- and time-dependent manner (**Figures 4A, B**). There was therefore a dose-dependent relationship between inflammation and viral infection. ELISA results showed an increase in IL-1β expression level in cell supernatants, but there was no significant difference in IL-18 level compared to that in uninfected cells (**Figure 4C**). Liver and lymphocyte samples collected from 1-day-old chicks were used to examine the levels of these two key cytokines (**Figures 4D–F**). IL-1β and IL-18 mRNA expression levels were elevated in both liver and lymphocytes. These results confirmed the YT strain–induced inflammatory response *in vitro* and *in vivo*.

### 3.6 NF-κB and MAPK signaling pathways are involved in the regulation of YT strain–induced inflammatory signaling pathway

**Figure 5:**
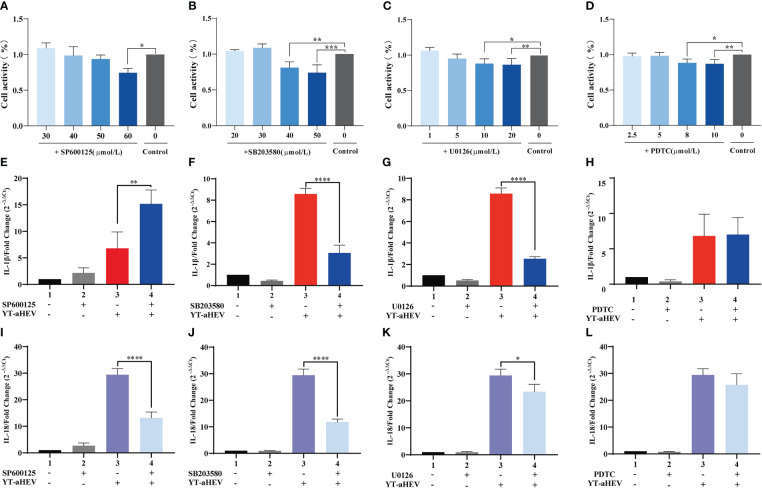
The effects of MAPK and NF-κB signaling pathway on YT strain–induced inflammatory response in LMH cell. **(A–D)** CCK8 detects the effects of different inhibitor concentration on LMH cells. **(E–H)** Effect of MAPK inhibitors (U0126, SB 203580, and SP600125) and NF-κB (PDTC) on IL-1β. **(I–L)** Effect of MAPK inhibitors (U0126, SB 203580, and SP600125) and NF-κB (PDTC) on IL-18. Symbols indicate inhibitor-treated and untreated cells; *P < 0.05, **P < 0.01, ***P < 0.001 and ****P < 0.0001.

The MAPK signaling pathway is associated with the induction of inflammatory factors. The NF-κB transcription factor is the master regulator of inflammation and immune homeostasis. Many important pro- and anti-inflammatory factors are transcriptionally regulated. Therefore, we focused on whether MAPK signaling pathways and NF-κB are involved in YT strain–induced inflammation. For this purpose, we compared the levels of IL-1β and IL-18 in inhibitor-treated and untreated cells. Signaling pathways included p38 MAPK, JNK, p44/42 MAPK (ERK1/2), and NF-κB. In our experiments, the optimal concentrations of different inhibitors were used including SP600125 (a JNK inhibitor, 50 μmol/L), SB 203580 (a p38 inhibitor, 30 μmol/L), U0126 (an ERK1/2 inhibitor, 5 μmol/L), and PDTC (NF-κB, 10 μmol/L). They were selected on the basis of the recommended range of concentrations and their actual toxic effects on responder LMH-cell viability and proliferation (**Figures 5A–D**). The cells were treated with the inhibitors for 2 h, followed by viral infection. The intensity of the inflammatory reaction was evaluated using the mRNA expression levels of IL-1β and IL-18 and other cytokines at 6 hpi. This particular time was chosen because YT strain infection had induced a strong inflammatory response in previous viral infection experiments (**Figures 4A, B**). The mRNA expression level of IL-1β and IL-18 in inhibitor-treated and untreated cells showed remarkable differences after the addition of P38, JNK, and ERK signaling pathway inhibitors. Specifically, the level of IL-1β mRNA expression was increased after SP600125 (JNK inhibitor) treatment (**Figure 5E**), whereas the level of IL-18 mRNA expression was significantly decreased (**Figure 5I**). The expression levels of both interleukins were downregulated after treatment with the inhibitors of SB 203580 (**Figures 5F, J**) and U0126 (**Figures 5G, K**); however, there were no significant changes in the mRNA expression levels of IL-1β and IL-18 in the PDTC-treated group (**Figures 5H, L**).

### 3.7 Inflammasome complexes are involved in the inflammatory response induced by YT strain infection

**Figure 6:**
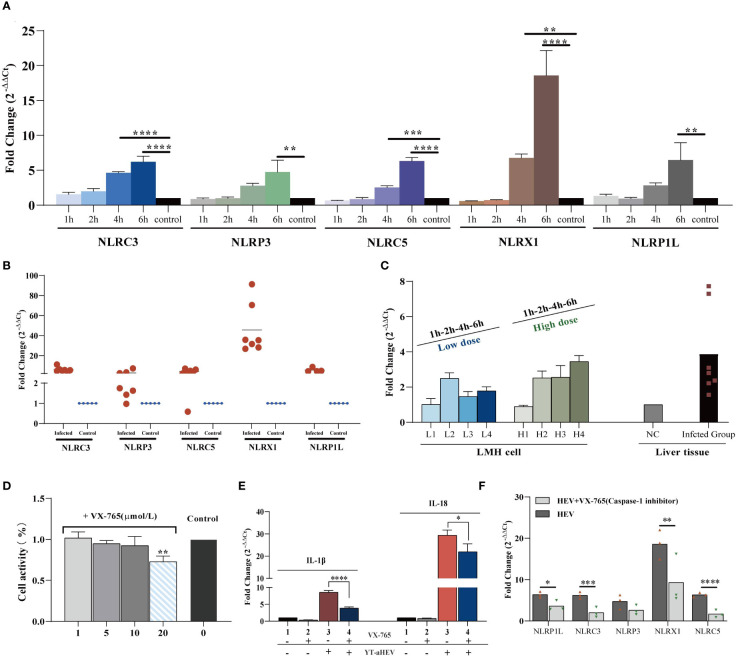
YT strain–mediated inflammation is caspase-1 dependent. **(A)** Transcriptional levels of NLRs in the LMH cell. LMH cells were infected with YT strain for 1, 2, 4, and 6 h. **(B)** Transcriptional level of NLRs in the liver tissue. **(C)** Transcriptional level of caspase-1 in the liver or LMH cell from the YT strain infected. LMH cells were infected with YT strain for 1, 2, 4, and 6 h. Two different initial infection doses were used: a high infection dose = 300 TCID_50_ and a low infection dose = 150 TCID_50_. The induction of caspase-1 was analyzed using real-time PCR and normalized to the value for β-actin. **(D)** CCK8 detected the effects of different concentration of VX-765 (a caspase-1 inhibitor) on LMH cell. **(E)** YT strain infection induced IL-1β and I-18 was produced dependent on caspase-1. **(F)** Consistent with the decreased caspase-1 activity, NLRs activity was attenuated in VX-765 treated LMH cells. Symbols indicate inhibitor-treated and untreated cells; *P < 0.05, **P < 0.01, ***P < 0.001 and ****P < 0.0001.

The term “inflammasome” refers to large multiprotein complexes that sense intracellular danger signals *via* NOD-like receptors (NLRs). The nomenclature of inflammasomes is based on the NLR, which is a family of PRRs. TLR activation, which results in pro–IL−1β transcription and translation; and NLR−induced IL−1β processing and release *via* a caspase−1–dependent mechanism are both required for IL−1β release from primary macrophages (34). Inflammasomes are multiproteins oligomers formed in response to a wide-range of stimuli, resulting in the recruitment of procaspase-1 and its subsequent cleavage into enzymatically active caspase-1 (35). To investigate inflammasome activation in LMH cells and the 1-day-old chicks, the mRNA expression levels of possible NLRs and caspase-1 were determined. To date, five types of avian inflammasomes have been identified: NLRC3, NLRP3, NLRP5, NLRX1, and NLRP1L. The expression levels of NLRC3, NLRC5, and NLRX1 increased significantly as early as 4 hpi. A significant increase in NLR activity was observed in the LMH cells at 6 h when compared to uninfected control cells (**Figure 6A**). Evaluation of the relative expression levels in the liver samples of uninfected chickens demonstrated a large upregulation of all NLRs (**Figure 6B**). These results suggest that inflammasomes play a role in the regulation of inflammatory signaling pathways induced by aHEV infection. NLRX1 was the most significantly upregulated gene *in vitro* and *in vivo*. Caspase-1 mRNA expression was assessed, and the results indicate that it was upregulated *in vitro* or *in vivo* and that it had a dose-dependent association with viral infection in LMH cells (**Figure 6C**). VX-765, a potent and selective caspase-1 inhibitor, can effectively inhibit the release of IL-1β and IL-18 by inhibiting caspase-1 activity. An *in vitro* experiment was performed to determine the possible inhibitory effect of VX-765 on the inflammatory response. LMH cells were treated with caspase-1–specific inhibitor VX-765 (10 μmol/L; **Figure 6D**) for 2 h. LMH cells were then infected with YT strain at a dose of 300 TCID_50_, and the cells were collected 6 h after infected. We found that the expression levels of NLRP3, NLRC3, NLRC5, NLRX1, NLRP1L, IL-1β, and IL-18 decreased with inhibitor treatment. We therefore determined the activation of the inflammatory pathway is dependent on caspase-1 (**Figure 6E**). Consistent with the decreased caspase-1 activity, NLR activity was attenuated in VX-765 treated LMH cells (**Figure 6F**).

### 3.8 With inhibitors of the NF-κB and MAPK signaling pathway deregulated inflammasome signaling

**Figure 7:**
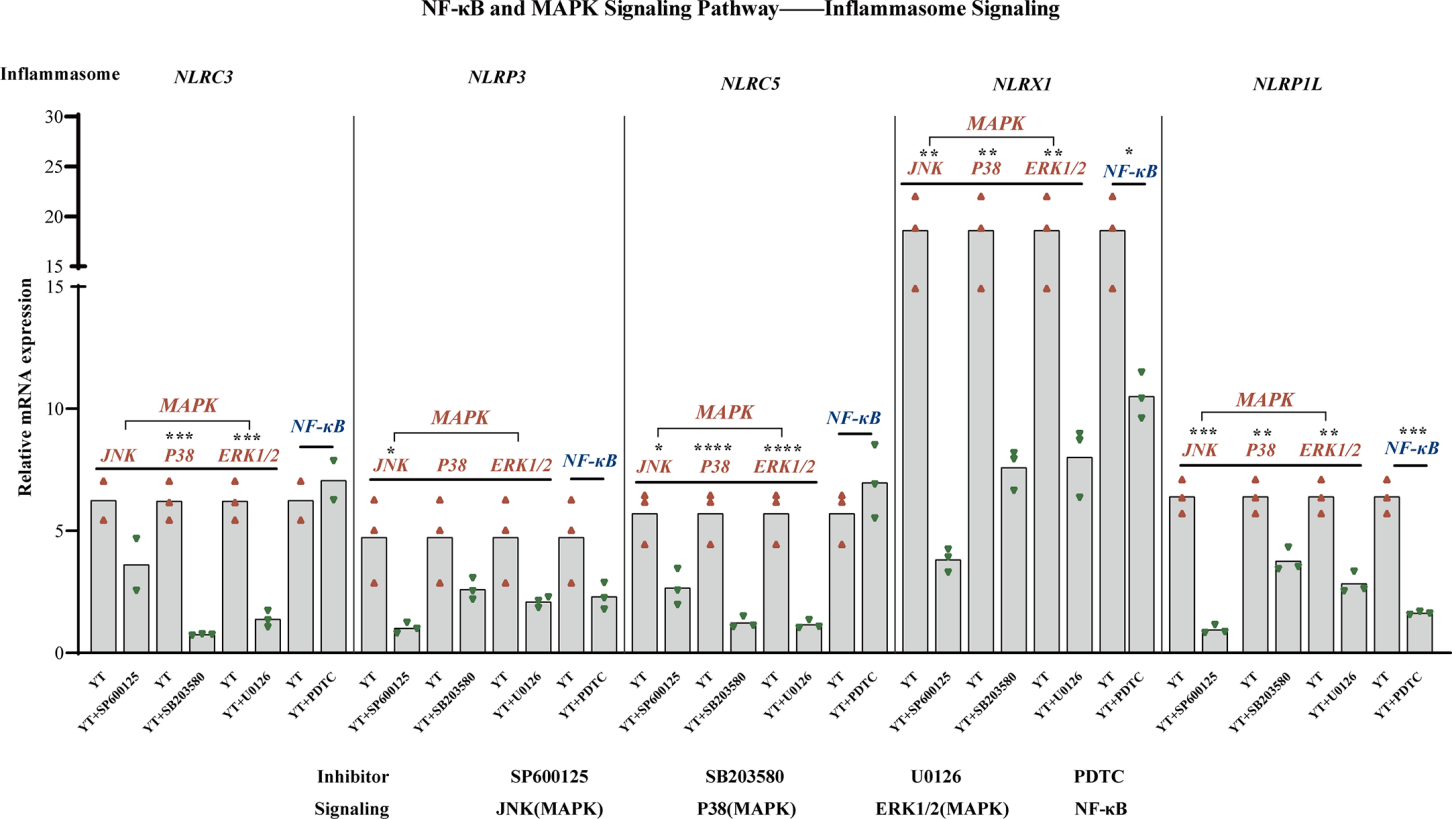
MAPK (JNK, P38, and ERK1/2) and NF-κB are involved in NLRs production. NLRX1 expression was suppressed by treatment with SP600125 (not significant), SB 203580 (P < 0.01), and U0126 (P < 0.01). NLRP3 is regulated positively by SP600125 (P < 0.05), SB 203580 (not significant), U0126 (not significant), and NF-κB (not significant). NLRC5 expression was suppressed under the treatment of SP600125 (not significant), SB 203580 (P < 0.01), and U0126 (P < 0.01). NLRX1 activity is attenuated in consistent with the decreased of JNK (P < 0.01), P38 (P < 0.01), ERK1/2 (P < 0.01), and NF-κB (P < 0.05). NLRP1L activity is attenuated consistent with the decreased of JNK (P < 0.01), P38 (P < 0.01), ERK1/2 (P < 0.01), and NF-κB (P < 0.01). The mRNA expression levels of NLRs were measured using qRT-PCR and calculated in relation to the expression level of β-actin. Symbols indicate comparisons with the control group; *P < 0.05, **P < 0.01, ***P < 0.001 and ****P < 0.0001.

During inflammatory processes, phosphorylation of MAPKs (JNK, ERK, and p38) plays a critical role by triggering the regulation of NF-κB signaling pathway. Emerging experimental evidence indicates that both MAPK and NF-κB play important roles in NLRP3 activation (36, 37). For example, the P38 MAPK signaling pathway can mediate NLRP3 upregulation by significantly increasing caspase-1 cleavage (38). The mRNA levels of several inflammasomes were examined to investigate whether the activation of NLRs inflammasomes could be suppressed by inhibiting MAPK/NF-κB signaling throughout YT strain infection **Figure 7**. LMH cells were treated with inhibitors of JNK (SP600125), P38 (SB 203580), ERK1/2 (U0126), and NF-κB (PDTC) signaling pathways, respectively, and, following incubation with the YT strain, cells were harvested 6 hpi. Previously, we found that all five inflammatory pathways were involved in the validation response induced by YT infection, and we therefore examined them using qRT-PCR. Treatment with PDTC inhibitor downregulated NLRP1L (P < 0.01) and NLRX1 (P < 0.05) expression in LMH cells compared to that in untreated LMH cells. JNK and p38 were suppressed using JNK, p38, and ERK1/2 inhibitors to verify whether MAPK was involved in inflammasome activation. JNK positively regulated NLRP1L (P < 0.01), NLRP3 (P < 0.05), NLRX1 (P < 0.01), and NLRP5 (P < 0.05) but was not associated with NLRC3. These results suggest that the P38 ERK signaling pathway may positively regulate NLRP1L (P < 0.01), NLRC3 (P < 0.01), NLRX1 (P < 0.01), and NLRP5 (P < 0.01) and was not associated with NLRP3.

## 4 Discussion

HEV is a causative agents of viral liver infections. In addition to humans, HEV strains have been genetically identified in swine, chickens, sika deer, mongooses, and rabbits (39). However, it is well known that aHEV is difficult to isolate and culture *in vitro*, which poses a major obstacle to the in-depth study of this virus. The factors contributing to the difficulty of *in vitro* culture are complex and unknown. However, on the basis of relevant studies (12), we speculate that difficulties in replication are related to virus–host interactions, such as the host innate immune response induced by the viral infection. The role of innate immune responses in aHEV infection has also not been extensively studied. In this study, one aHEV strain associated with chicken liver swelling and hemorrhage in China was obtained and designated as the YT strain. This virus could efficiently replicate in LMH cells. In addition, animal infection experiments demonstrated that this strain could infect SPF chick embryos and chicks *via* intravenous inoculation. All the above findings provide a basis for this study.

To characterize its pathogenic potential, we used the YT strain to inoculate SPF chicken embryos. Viral RNA was detected in plasma (12/14), fecal (13/14), and liver samples (13/14). The animal experiments presented in the current study provide evidence supporting the relationship between liver damage and viral invasion from several aspects, including gross lesions, histological lesions, the liver index, and ALT and AST levels.

A growing body of evidence indicates an active response of the host innate immunity to HEV infection, both in experimental models and in patients (14). In this study, we found that the YT strain efficiently induced high levels of OASL in the liver and LMH. Viral infection induces a more intense Mx response in the liver than in LMH cells. In addition, MDA5 and inflammation-related cytokines were activated *in vivo*. Notably, MAVS mRNA levels decreased in LMH cells and the liver. MAVS is a key factor driving innate immunity against RNA viruses, and viruses have evolved a number of antagonistic strategies against MAVS to avoid detection by the host immune system. The hepatitis virus family is a representative example of an antagonistic strategy (40). As the only decrease in antiviral proteins follows viral infection, the mechanism by which MAVS regulates the innate immune response to the YT strain merits further research.

Viral infections typically induce inflammatory reactions. IL-1β is a pro-inflammatory cytokine and a central regulator of inflammation that binds to the IL-1 receptor to exert its broad biological effects. IL-18 is a pleiotropic cytokine that that is required for activated macrophages, natural killer cell, and T cells to produce IFN-γ (41). Both IL−1β and IL−18 are recognized for their ability to cause a wide variety of biological effects associated with infection, inflammation, and autoimmunity (42). We used IL-1β and IL-18 activation as indicators of inflammatory pathway activation and used fluorescent qRT-PCR or ELISA to detect the expression of both cytokines after YT infection of LMH cells and SPF chickens. LMH cells infected with the YT strain significantly increased the intracellular levels of IL-1β and IL-18 in both a dose- and time-dependent manner. *In vivo* dates support this conclusion: IL-1β and IL-18 are upregulated in both in lymphocytes and the livers of infected chickens.

TLR activation is important in microbial infections and is an important part of the immune system. TLRs are abundantly expressed in hepatocytes, the primary targets of HEV. We found that, both *in vitro* and *in vivo*, the YT strain activated several TLRs, including TLR3, TLR4, TLR5, TLR7, TLR1, and TLR21. The expression of TLRs at the gene level in aHEV individuals or LMH cells was compared with that in healthy individuals or LMH cells. TLR3 expression in liver tissue was significantly upregulated in YT strain–infected chickens, and it was the most upregulated of several TLRs. It has been shown that overexpression of TLR3 inhibits HEV replication in cell culture models. Blocking the TBK1/IKKϵ complex and TLR3 downstream molecules consistently increases HEV replication (43).

TLR ligands activate various downstream intracellular signaling cascades, such as the NF-κB and MAPK pathways, ultimately leading to the production of inflammatory mediators. We used four available MAPK signal pathway inhibitors: the JNK pathway inhibitor, SP600125; the p38 pathway inhibitor, SB 203580; the ERK pathway inhibitor, U0126); and the NF-κB pathway inhibitor, PDTC; to investigate the contribution of each MAPK signaling pathway and NF-κB to YT strain infection *in vitro*. When the MAPK pathway was effectively blocked by SP600125 and SB 203580, the expression level of IL-18 decreased markedly. When the JNK pathway was effectively blocked, the expression level of IL-1β increased. In the presence of SB 203580 or U0126, the expression level of IL-1β decreased markedly. We note that PDTC did not affect the expression level of either IL-1β or IL-18. In conclusion, we have illustrated that infection with the YT strain can activate the MAPK pathway.

Inflammasomes are intracellular multiprotein complexes that mediate the proximity-induced autoactivation of caspase-1 (44). IL−1β and IL−18 are usually required for caspase-1–mediated cleavage and full activation and secretion. We examined the expression of caspase-1 in LMH cells and livers infected with the YT strain using VX-768 to inhibit the activity of capase-1. In this study, we identified five kinds of avian inflammasomes involved in the YT strain infection in a dose- and time-dependent manner. In the mitochondrial NLR family, member X1 (NLRX1) was the most upregulated gene. Conflicting reports have been presented regarding the role of NLRX1 in antiviral responses (45). Therefore, the function of NLRX1 in the defense of chickens against aHEV requires further in-depth study. The activation of caspase-1, IL-1β, and IL-18 in the liver or LMH cells following, with or without VX-765 (a caspase-1 inhibitor), revealed a strong correlation between caspase-1 activation and virus induced inflammation.

The study of aHEV pathogenesis has become a necessity due to the increasing rate of clinical infection. The ultimate goal is to provide a more theoretical basis for the prevention and control of aHEV through more extensive, systematic, and in-depth studies. With regard to the results of this study, *in vivo* and *in vitro* infection experiments identified signaling pathways and dysregulated expression of genes involved in the inflammatory response induced by aHEV. Infection with YT strain leads to liver injury and could activate inflammation associated with the TLR–JNK–NF-κB pathway and caspase-1 inflammasomes. These findings provide important insights regarding the liver trauma–related innate immune response of the liver, the cellular basis of cytokine production in the liver, and inflammation-induced liver diseases. These pathways and key genes provide potential targets for the prevention and control of this disease. In addition, aHEV can be transmitted by the fecal–oral route, so strict biosecurity measures on poultry farms can help to limit the spread of the virus. Pathogen detection is also an important part of animal disease prevention and control, and it is important to continually improve and optimize detection techniques to maximize the benefits of pathogen detection. However, it is important to note that, although new genotypes of aHEV have been reported since 2016, the pathogenicity of classical genotype should not be ignored. The genotype 3 strain in this study proved to be still present in the flock and caused significant liver damage.

In conclusion, aHEV is a pathogen that poses a serious risk to the poultry industry, but many questions remain unclear. The development of strict biosecurity regulations at chicken farms, the elimination of positive individuals and further studies on pathogenic mechanisms of the virus will all contribute to the control of aHEV infection.

## Data availability statement

The original contributions presented in the study are included in the article/supplementary material. Further inquiries can be directed to the corresponding author.

## Ethics statement

The animal study was reviewed and approved by Shandong Agricultural University Animal Care and Use Committee (SDAUA-2016–002).

## Author contributions

YZ conceived and performed the experiments, analyzed the data, and drafted the manuscript. ZNC contributed to the experimental techniques and materials. SC and YW edited the manuscript. ZZC give guidance on the ideas. PZ provided leadership of this subject and supervised the project. All authors contributed to the article and approved the submitted version.
